# Daniel E. Lieberman Exercised: Why Something We Never Evolved to do Is Healthy and Rewarding

**DOI:** 10.1093/emph/eoaa040

**Published:** 2020-10-28

**Authors:** B N Horowitz

**Affiliations:** e1 Harvard Medical School, Harvard-MIT Health Sciences & Technology, Boston, MA, USA; e2 Department of Human Evolutionary Biology, Harvard University, Cambridge, MA, USA; e3 Division of Cardiology, David Geffen School of Medicine at UCLA, Los Angeles, CA, USA 11 Divinity Avenue, Room 47, Peabody Museum, Cambridge, MA 02138, USA

Soon, German dog-owners will be legally required to walk their dogs for a total of at least an hour each day. The most pampered Pomeranian, though it has grown used to couches and carpets, must now be allowed to answer the call of the wild by moving its little body through the park. The new *Hundeverordnung* (‘Dog Act’) recognizes that not only every hound but even a skinny, shaking Chihuahua contains, somewhere deep inside, an ancestral wolf.

Of course, the wolf, unlike the Chihuahua, didn’t need government-mandated doses of exercise. To survive, the wolf hunted, and to hunt, the wolf ran. Yet, its great-great-grand-pup, the domesticated dog, must be regularly and intentionally exercised to maintain its health. This is no less true for the domesticated human, as Daniel Lieberman explains in his myth-busting and fascinating new book, *Exercised: Why Something We Never Evolved to Do Is Healthy and Rewarding*.

Lieberman uses both anatomical and anthropological evidence to show how our speed, strength, and endurance developed to help us forage, fight, and hunt—activities our forebears had no choice but to do on a regular basis. Chasing down antelope, trekking long distances for wild plants and occasionally outrunning predators ensured the propagation of our genes. Thus, what is now called ‘physical fitness’ was once closely aligned with what scientists call ‘evolutionary fitness’—whether an organism is sufficiently adapted to its environment to survive and reproduce. Today, however, most of us have no urgent need to run down a meaty ungulate or walk ten miles to dig up tubers. Calories are plentiful and chairs abound.

So why move? Isn’t exercise a slap in the face of all our hard-won human progress, of civilization itself? Well, coming from a cardiologist, my answer may not be a surprise: Regular, vigorous, sustained activity can save your life! But my experience with patients—and with myself—shows that a wide gulf separates knowing and doing.

In fact, Lieberman explains, there’s a good reason so many of us view the gym as a house of horrors designed by a master torturer. Spoiler alert: It’s evolution’s fault. For most of human existence, calories were scarce and exertion inevitable, so there was little need for natural selection to incentivize exercise by making it, you know, feel good.

Some will be disappointed to learn that the book doesn’t stop with this ironclad excuse for getting out of gym class (signed by evolution itself!). Instead, Lieberman goes on to explain how and why exercise, that flagrant waste of calories and of civilization’s labor-saving advances, is nonetheless critical to improving our health and to combating aging, and even how we might trick ourselves into doing it.

It would seem difficult to deliver this message in a way that doesn’t feel both familiar and insufferable, but Lieberman manages, partly because of his writing’s warmth and empathy. That empathy springs from his own lived experience of the old conundrum: we know we should exercise, but we don’t want to.

Once a scrawny kid picked last for teams, Lieberman is now an accomplished marathon runner as well as a popularizer of barefoot running (he features prominently in the bestselling book *Born to Run*). He has even outrun horses in an endurance race over mountains! But he doesn’t boast about his athletic prowess, like those ostentatiously grunting gym bros who seem to be exercising not *near* you but *at* you. In fact, Lieberman has a term for them, and for the ‘runners’ who look down their noses (or behind their backs) at mere ‘joggers’, and for the rest of their arrogant ilk: exercists. Unlike the holier-than-thou exercists, who view a lack of fitness as a moral failing, Lieberman is at pains to normalize not wanting to exercise.

Another reason Lieberman can make his basic message (exercise is good for you, try to do it more) sound new is because he approaches the topic from a new angle—or rather a very old one. As an evolutionary biologist and anthropologist, he has long studied how the human body evolved in tandem with certain behaviors. He uses these two lenses—how humans behaved and how they evolved—to put into perspective the exhaustive, and exhausting, the literature on exercise.

For instance, rather than join the chorus lamenting how Westernized people often fail to get the optimal 8 h of sleep, he looks at observational studies questioning whether 8 h actually *is* optimal. He finds that not only do most Westerners average seven hours a night, but that hunter-gatherer people average even less (6.5–7), and that numerous studies have found higher mortality rates for people who sleep less *or more* than seven hours on average. He repeatedly offers this sort of careful and clear evaluation of health and exercise studies that can be challenging to interpret for not only patients but many physicians as well.

As the ‘eight hours a night’ example suggests, the book often proceeds by debunking widely held beliefs. For example, were our hunter-gatherer ancestors actually huge, muscled strongmen, as many evangelists of paleo-fitness plans like CrossFit argue? Of course not, Lieberman says. Look at contemporary hunter-gatherer people like the Hadza of Tanzania, the San of the Kalahari, or the Aché of the Amazon—they are certainly fit, but also lean, and unlikely to beat Dwayne ‘The Rock’ Johnson in an arm-wrestling contest. Again, this makes sense in environments where energy is at a premium, since big muscles are *expensive*—not in terms of gym memberships, but calorically. To maintain his impressive physique, the Rock puts away more than *5000 calories a day*, spread across seven meals. On average, he eats 12 eggs and more than two pounds of cod per day—a diet he probably couldn’t maintain for long in our ancestors’ hardscrabble environments.

As a physician and science writer myself, I was deeply impressed by Lieberman’s straightforward synthesis of a mass of research on human health and exercise that is messy, confusing, and often self-contradictory. He cuts through the morass of studies (some large, some small; some randomized and controlled, some population surveys; some conducted over months, others over decades) to overturn conventional wisdom and offer readers (including this cardiologist) unusually digestible insights.

A 339-page book could seem like a marathon itself, but these pages never plod. The many illustrative stories are one big reason why. Drawn from different eras and areas, they consistently provide historical, cultural, and sometimes scientific context for understanding important concepts. Sometimes he flexes his literary muscles with references to the co-sleeping arrangements of Ishmael and Queequeg in *Moby Dick*, or to the all-night balls of Jane Austen (which he compares to the endurance dancing of the San, Hadza and Tarahumara peoples).

Anecdotes from his own experience do a lot of heavy lifting, too. Writing on whether sitting is unhealthy, he recalls with a shiver days he spent sitting on a sled jouncing across Greenland in −30˚F cold. On the subject of sprinting, he bravely admits how he set his own personal speed record—ingloriously dashing away from a hyena who wandered up to him in Kenya. (It might seem merely an amusing tangent, but he then uses it to explain differences in speed and stamina that result from physiological variation across species.) These moments add to his arguments both self-effacing humor and a first-person authority.

Another unexpected but very welcome feature of the book is the consistent inclusion of women’s experiences and health issues. (He may have been influenced here by the example of his mother, a jogger who campaigned in the 1960s for the University of Connecticut to open its athletic facilities to women.) Historically, such attention to women has been all too rare in the scientific and medical literature. For instance, until the NIH Revitalization Act was passed in 1993, NIH-funded studies were not required to include women. As a result, for decades all we knew about heart disease was really only what we knew about *males* with heart disease.

Thanks in part to the swelling ranks of female researchers, this imbalance is slowly being improved. Admirably, Lieberman is putting his shoulder to the wheel, too. In fact, the book begins with him schlepping a treadmill to remote Pemja, Kenya, to study the running and walking movement of Kalenjin women. When describing the Tarahumara endurance races in Mexico (made famous by *Born to Run*), he begins with the *ariwete*, the women’s event. Again and again, whether in Mexico, Kenya, Tanzania or Ethiopia, he turns his attention to movement and athleticism among both men and women.

One powerful example of Lieberman’s balanced approach to the genders is his proposed expansion of the so-called Grandmother Hypothesis to the more inclusive and accurate Active Grandparent theory (suggesting both male and female elders labored to take care of their children’s children). Without fanfare, he upends a gendered assumption and simultaneously presents a mechanism by which activity in later life may promote longevity.

Lieberman’s concern for differing experiences is evident throughout the book, as his lack of interest in one-size-fits-all prescriptions for exercise. He asks at one point, ‘Do we really expect the same action plan to work for people who are overweight or thin, shy or outgoing, insecure or confident, men or women, college graduates or less educated, rich or poor, urban or rural, stubborn or docile?’ Instead, he distills the research on exercise’s benefits so different people can use it as they see fit.

Interestingly, that German dog-walking law that requires dogs to get an hour of exercise per day already faces backlash due to its inflexibility. People are complaining about the lack of exceptions for old or sick dogs, or pointing out that an hour may be too long for some dogs to spend outside on hot days. Those German legislators—exercists for dogs—might have benefited from reading *Exercised*, and its clear-eyed approach to a complex subject.

In particular, they might have learned something from Lieberman’s proposal to make physical education mandatory at universities. Though he generally favors a carrot-and-stick approach over coercion, he makes an exception for college, where students are still developing lifelong habits. Even here, though, his proposal takes into account the wide range of human abilities by offering flexible options—unlike the *Hundeverordnung.*

In treating the complicated topic of human fitness, Lieberman has managed to demystify, and demythify, the swirl of evidence about exercise’s benefits and risks. While careful not to be explicitly prescriptive, *Exercised* uses empathy, humor, storytelling and careful reasoning to help readers come to their own conclusions. Understanding how and why we moved in our ancestral past can help us think more clearly about how and why we should move now. If we’re lucky, it won’t involve hyenas.

